# College and Hospital Notes

**Published:** 1899-01

**Authors:** 


### COLLEGE AND HOSPITAL NOTES.

The hospital corps of the 74th Regiment, N. G. N. Y., will give an exhibition drill in litter work, at the 74th Regiment Armory, on Virginia street, Thursday evening, January 5, 1899. It will be remembered that this corps, although not in active service during the late Spanish-American war, did much to alleviate the sufferings of its fellow-soldiers. August 30, 1898, at the time of the arrival of the sick train of the 65th Regiment, N. Y. V., the corps distinguished itself by successfully unloading 108 men, mostly bedridden, in less than forty-five minutes. In communications addressed to the regimental surgeon about that time, His honor Mayor Conrad Diehl, Dr. Herman Mynter, Dr. William H. Heath, Dr. Joseph T. Cook, Dr. Dewitt Sherman and many other prominent physicians actively engaged in caring for the sick men, expressed themselves in exalted terms as to the work performed. Acting Assistant Surgeon N. W. Wilson, U. S. Army, of Fort Porter, who accompanied the train in transit to Buffalo, congratulated the corps and made the statement that "at Dunn Loring the loading of the cars occupied an hour and a half, while the unloading at Buffalo was accomplished in considerably less than half that time, and was carried out with less friction and immeasurably more comfort to the patients." Tickets for this drill are now on sale at H. Kleinhans & Co.'s store.

---

THE Southern Medical College Association held its seventh annual meeting at Memphis, Tenn., December 5, 1898. The chief business of importance transacted was the unanimous adoption of the four-year course by the colleges forming the association. This is to go into effect January 1, 1899. This change has been discussed at the two preceding meetings, but action has been deferred until now. The adoption of the four-year course will necessitate certain amendments to the constitution and by-laws of the association, but these cannot be acted upon until the next meeting. The following officers were elected for the ensuing year: Dr. G. A. Ketchum, of the Alabama Medical College, president; Dr. Christopher Tompkins, of the Medical College of Virginia, vice-president; Dr. G. C. Savage, of the Vanderbilt University, secretary and treasurer. The next meeting of the association will take place at New Orleans, at the time of meeting of the Southern Surgical and Gynecological Association, November, 1899.

---

A CONSUMPTION hospital or state sanitarium for consumptives is under contemplation in this state. The Brush committee of the Senate, composed of Senators George W. Brush, George A. Davis and Frank Gallagher, which is considering the advisability of establishing such an institution in the Adirondacks, held a meeting in the library of the City Hall, at New York, December 5, 1898.

Prof. Evans, Dr. J. H. Pryor, of Buffalo, the originator of the plan, and Dr. J. H. Raymond, all experts on pulmonary diseases, appeared before the committee and testified in favor of the establishment of such a hospital on account of the natural advantages afforded by the climate of the Adirondacks.

We trust the committee will report progress to the legislature early in the next session.

---

A CANCER hospital is under discussion for establishment in Buffalo. Assemblyman Fred Nixon, of Chautauqua county, who is probably to be the next speaker of the Assembly, was in Buffalo recently, and had a conference with Dr. Roswell Park concerning the advisability of establishing such an institution here. It is understood that Mr. Nixon is favorably impressed with the idea, and a number of prominent Buffalo men are doing what they can to promote it.

Last winter an appropriation of $10,000 was made by the legislature for the purpose of enabling the Buffalo University to make certain laboratory experiments in the line of treating cancer.

A further sum of $25,000 will probably be asked for this winter to equip the hospital. The institution will be conducted on the plan of the German Cancer Hospital at Berlin. In that country physicians immediately send to the hospital any cases which they are in doubt about and in that way much suffering is averted and many lives are saved.

---

THE Ohio State Board of Medical Registration and Examination, October 4, 1898, adopted the following resolution :

*Resolved*, That after October 4, 1898, no medical college or university will be recognised as in good standing by this board which shall graduate in one term students who have taken but two terms of a four years' course, and applicants presenting such credentials will not be entitled to registration in Ohio.

---

THE German Hospital staff at its last meeting elected the following officers for the current year : President, Dr. Max Breuer; vice-president, Dr. Charles Weil; secretary, Dr. H. G. Bentz; house committee, Dr. S. Goldberg, Dr. W. Meisburger and Dr. Marcell Hartwig.

The corner-stone of the new building was laid early in December in the presence of a large assemblage of citizens and the ceremonies were appropriate and interesting. The development of this institution is watched with greatest interest by many philanthropic and public-spirited men and women. Its promoters are men of energy and its success cannot be doubted.

At the University of Buffalo some changes have lately occurred in the faculty. Dr. Charles Cary has resigned a portion of his chair, materia and therapeutics, retaining his professorship of clinical medicine. Dr. Eli H. Long has been appointed professor of materia medica and therapeutics.

